# Preservation of Genetic Variation in a Breeding Population for Long-Term Genetic Gain

**DOI:** 10.1534/g3.120.401354

**Published:** 2020-06-08

**Authors:** David Vanavermaete, Jan Fostier, Steven Maenhout, Bernard De Baets

**Affiliations:** * KERMIT, Department of Data Analysis and Mathematical Modelling, Ghent University, B-9000 Ghent, Belgium,; ^†^ IDLab, Department of Information Technology, Ghent University - imec, B-9052 Ghent, Belgium, and; ^‡^ Progeno, B-9052 Ghent, Belgium

**Keywords:** Genomic Prediction, Genetic Gain, Plant Breeding, QTL Fixation, Simulation Study, GenPred, Shared data resoureces

## Abstract

Genomic selection has been successfully implemented in plant and animal breeding. The transition of parental selection based on phenotypic characteristics to genomic selection (GS) has reduced breeding time and cost while accelerating the rate of genetic progression. Although breeding methods have been adapted to include genomic selection, parental selection often involves *truncation* selection, selecting the individuals with the highest genomic estimated breeding values (GEBVs) in the hope that favorable properties will be passed to their offspring. This ensures genetic progression and delivers offspring with high genetic values. However, several favorable quantitative trait loci (QTL) alleles risk being eliminated from the breeding population during breeding. We show that this could reduce the mean genetic value that the breeding population could reach in the long term with up to 40%. In this paper, by means of a simulation study, we propose a new method for parental mating that is able to preserve the genetic variation in the breeding population, preventing premature convergence of the genetic values to a local optimum, thus maximizing the genetic values in the long term. We do not only prevent the fixation of several unfavorable QTL alleles, but also demonstrate that the genetic values can be increased by up to 15 percentage points compared with truncation selection.

In times of climate change and rapid population growth, new methods need to be developed to further improve different crop properties like yield and resistance to pathogens and drought (Tester and Landridge 2010). These properties are controlled by different chromosomal regions or quantitative trait loci (QTL), making it difficult to improve crop properties by only using phenotypic characteristics (Dekkers and Hospital 2002). Initially, pedigree information was used to guide the selection of parental lines in animal and plant breeding. Nowadays, molecular markers like single nucleotide polymorphisms (SNPs) serve as proxies for QTL, assuming that markers are in strong linkage disequilibrium with one or more QTL (de Roos *et al.* 2008). The linear relationship between the genetic markers (genotype) and the phenotype can then be estimated using a mixed effects model. This concept was first introduced in marker-assisted selection (MAS), but only minor improvements in yield were reported (Goddard and Hayes 2002). Genomic selection was introduced as an alternative for MAS (Meuwissen *et al.* 2001). By using markers that cover the complete genome, the fraction of the genetic variance that can be explained by the molecular markers was better captured, leading to an improved estimation of large and small QTL effects (Heffner *et al.* 2009, 2010; Beyene *et al.* 2015). Genomic selection improved yield in animal and plant breeding and reduced the time in between breeding cycles (Hayes *et al.* 2009). For example, crops like oil palm (Elaeis *guineensis Jacq*.) reach sexual maturity after three years but require 13 to 15 years before phenotypic characteristics can be obtained. The transition of phenotypic selection to genomic selection reduced the time of one breeding cycle from 15 to three years (Cros *et al.* 2018). In time, genomic selection has further evolved and has become a powerful tool in animal and plant breeding (Meuwissen *et al.* 2001; Bernardo and Yu 2007; Crossa *et al.* 2010). Over the last years, several advancements were achieved ranging from yield maximization to the development of new drought/heat-resistant plants (Wang *et al.* 2019; Sun *et al.* 2019; Suontama *et al.* 2019). Nevertheless, the implementation of genomic selection in certain breeding populations with complex traits and environmental interactions is still challenging (Juliana *et al.* 2018; Voss-Fels *et al.* 2018).

Several simulation studies on genomic selection have resulted in high prediction accuracies and genetic values in the short term (VanRaden *et al.* 2009; Hayes *et al.* 2009). These studies often rely on *truncation* selection of the parents, leading to a high genetic gain in the short term but the loss of favorable QTL alleles, genetic variation and prediction accuracy over time (Jannink 2010). Truncation selection selects the top fraction of the individuals based on their genomic estimated breeding values (GEBVs), which serve as estimators for the true breeding values. Because the GEBVs are calculated as the sum of the estimated additive marker effects, the contribution of favorable small-effect QTL can be concealed leading to their loss in the breeding population, thus reducing long-term genetic gain. The loss of those favorable QTL alleles could be reduced by weighting the marker effects of favorable low-frequency alleles more heavily, thereby safeguarding long-term gain (Jannink 2010; Liu *et al.* 2015). In recent years, different parental methods have been developed that aim to reduce the loss in genetic variation. This helps to increase the prediction accuracy and the genetic gain in the long term. To preserve genetic variation, the selection of closely related individuals should be avoided (Lindgren and Mullin 1997) or the inbreeding coefficient should be minimized (Brisbane and Gibson 1995). Although genomic selection uses GEBVs for parental selection, alternative score functions to guide the parental selection have been proposed. The *criterion of usefulness*, which takes into account the selection intensity, mean genetic value and genetic variance of the breeding population using Markov chain Monto Carlo simulations, has improved long-term genetic gain (Lehermeier *et al.* 2017). An alternative parental selection scheme was proposed based on the genomic optimal haploid value, selecting parents that optimize the genetic values of their offspring (Daetwyler *et al.* 2015). This method was further improved by simulating the meiosis between parental haploids, yielding an improved prediction of offspring. This, in turn, leads to a more accurate evaluation of the double haploids, thereby guiding the parental selection to further increase long-term genetic gain (Müller *et al.* 2018).

Over the last years, new mating designs have been proposed to further improve the parental selection and maximize the genetic gain in the short or long term. In a new mating design, the genetic variation is preserved by penalizing crosses between two parents with high coancestry (Cervantes *et al.* 2016). Moreover, long-term gain was further improved by also minimizing the rate of inbreeding and controlling the allele heterozygosity and allele diversity (Akdemir and Sánchez 2016). The introduction of an optimal mating design using a two-part plant breeding selection with rapid recurrent genomic selection reduced the drop in genetic diversity, thus maximizing the conversion of genetic variance into genetic gain (Gorjanc *et al.* 2018).

Although parental selection methods play a major role in the realization of long-term genetic gain, as long as those methods are based on GEBVs, the results will be influenced by the choice of the prediction model and the training panel design. Several training panel designs have been proposed although no significant difference was observed in the long term, as long as the training panel was systematically updated over time (Akdemir *et al.* 2015; Rincent *et al.* 2012; Neyhart *et al.* 2017).

In this paper, the *scoping* method is presented as a new parental mating scheme to reduce the loss of favorable QTL alleles by preserving the genetic variation and thus maximizing the genetic value in the long term. The scoping method combines genetic progression (truncation selection) and the preservation of the genetic variation of each marker in the breeding population. Based on the observation that two closely related individuals might contain a different rare marker allele, both individuals should be selected to preserve the genetic variation of both markers in the breeding population. Therefore, in contrast to other methods, the genetic relationship or inbreeding coefficient is not taken into account, but individuals are selected based on their genotype, ensuring the maximal selection of the different marker alleles and thus maximizing the genetic variance of their offspring. By doing so, we reduce the risk of premature convergence of the genetic values to a local optimum. Combined with truncation selection, the genetic progression is ensured in the short as well as in the long term. We benchmark our proposed scoping method against two existing selection strategies: the *population merit* method (Lindgren and Mullin 1997) and the *maximum variance total* method (Cervantes *et al.* 2016). Both methods try to maximize long-term genetic gain by preserving the genetic variation of the breeding population, whereas the scoping method preserves the genetic variation by maximizing the variation of each marker, the population merit method preserves the genetic variation by minimizing the average genetic relationship of the parental population. Both the scoping method and the maximum variance total method aim to maximize the genetic variation of the parental population, and thus are good candidates against which our proposed scoping method can be benchmarked.

## Materials And Methods

We adopt the base population and breeding scheme of Neyhart *et al.* (2017), making it possible to compare our results with truncation selection as reported by Neyhart *et al.* (2017). The base population consists of two datasets of North American barley (Hordeum *vulgare*) from the University of Minnesota (UMN) and the University of North Dakota (NDSU) counting respectively 384 and 380 six-row spring inbred lines with 1590 biallelic SNP loci. Recurrent selection is applied to the base population to simulate the later breeding cycles (see Breeding scheme).

In a simulation study, the scoping method, in which a parental selection method is combined with a new mating design, is proposed and compared with truncation selection with random mating. The scoping method tries to maximize the genetic values in the long term, while preserving the genetic variation of the breeding population. It aims to avoid the loss of positive-effect QTL alleles, preventing the convergence of the genetic values to a local optimum. Because this method might select extreme GEBVs, the Pearson correlation cannot be used to evaluate the selection method due to its sensitivity to outliers. Instead, the mean genetic value of the breeding population, calculated on the basis of the true breeding values, is used to measure and evaluate the genetic gain for each method. The mean genetic value of the top-10 individuals is also reported. Our method aims to maximize the genetic gain of the top-10 individuals, while the remaining individuals of the breeding population serve to preserve important genetic marker alleles in the breeding population.

### Breeding scheme

**Figure 1:**
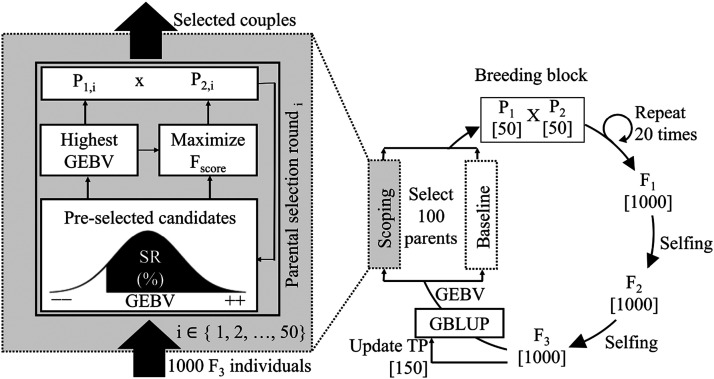
Overview of the recurrent selection scheme. First, 50 couples of parents ($P_{1}$, $P_{2}$) each produce 20 offspring yielding a total of 1000 F1 hybrids. Then, after two generations of single-seed descent, 1000 F3 individuals are obtained. From those F3 individuals, new parental lines are selected. Two different parental selection methods are considered: i) the baseline method selects 100 parents with the highest GEBVs (truncation selection); ii) the scoping method combines the selection of 50 parents ($P_{1}$) with the highest GEBVs and 50 parents ($P_{2}$) that maximize the genetic variation (see Equation (1)). After the parental selection, the TP is updated according to the tails method.

The recurrent selection scheme is illustrated in Figure 1. Starting with 100 individuals, a crossing block is constructed, coupling up the selected individuals. Each couple produces 20 offspring resulting in a total of 1000 F1 hybrids. After two generations of single-seed descent, 1000 F3 individuals are obtained. These individuals form the new breeding population from which again 100 parents are selected to start a new breeding cycle. This selection occurs either according to the *baseline*, population merit, population selection criterion, maximum variance total or scoping methods. The first breeding block (in breeding cycle zero) couples up 50 individuals of the NDSU dataset with 50 individuals of the UMN dataset with the highest phenotypic value, regardless of the parental selection method. This design choice ensures that each parental selection method has the same number of individuals in the breeding population over each breeding cycle. The subsequent parental selections are fully based on GEBVs, reducing the financial cost of phenotyping. A linear mixed effects model is used to obtain GEBVs from molecular marker scores (see Training panel). Each simulation consists of 50 breeding cycles and all results are averaged over 250 simulation runs.

### The baseline method

The baseline method selects 100 parents with the highest GEBVs (truncation selection) and couples them randomly. The idea is that favorable properties will be passed on to the next offspring, leading to high short-term gain and rapid fixation of favorable QTL alleles. However, several favorable QTL alleles will be eliminated from the breeding population during breeding, reducing long-term gain and causing the convergence of the genetic values to a local optimum.

### The scoping method

The scoping method continuously preserves genetic variation, avoiding premature convergence to a local optimum while ensuring a gradual increase of genetic values over breeding cycles. The parental selection is split into two parts: the pre-selection and the selection. First, a fraction of the breeding population with the highest GEBVs is pre-selected using truncation selection. This fraction, referred to as the scoping rate (SR), can take a value between 0.1 and 1. An SR of 0.1 pre-selects 100 individuals (10%) of the breeding population, whereas an SR of 1 will pre-select the entire breeding population (100%). During the selection, 100 different parents are chosen from the pre-selected population. In contrast to the baseline method, parents are not coupled randomly. From the pre-selected individuals, the one with the highest GEBV is chosen as the first parent. The second parent is chosen from the pre-selected individuals in such a way that the genetic variation of selected parents is maximized over each marker. Mathematically, the following score function is maximized:

$$F_{score}=\sum_{j=1}^{k}\text{var}\left(Z_{j,selection}\right)p_{j}$$ (1)

with *k* the total number of molecular markers, $Z_{j,selection}$ the incidence matrix of the selected parental genotypes (coded as -1, 0 and 1) at the *j*-th marker and $p_{j}$ a Boolean value. Initially, $p_{j}$ has a value of 1 for every marker position. When both alleles of a marker *j* are present in the selected population, the value $p_{j}$ is set to 0 before selecting the next couple of parents. Thereby, the score function will maximize the variance of the genotype over each marker for which both alleles are not yet present in the selected population, thus avoiding the loss of low-frequency marker alleles. If $p_{j}$ is 0 for all markers, the value of each $p_{j}$ is changed back to 1, again maximizing the variance over all the markers. At this moment, all the available marker alleles of the current breeding population are present in the selected parental population.

The scoping method combines truncation selection with a new mating design, coupling individuals with high GEBVs with individuals that maximize the genetic variation of their offspring. The pre-selection process avoids that individuals with lower GEBVs, which might maximize the genetic variation of certain parents, are not available for selection and thus avoids the loss of genetic gain in the short term. We expect that the mating design will reduce the loss of marker alleles while the pre-selection will eliminate unfavorable QTL alleles over time. This should lead to a slower but more accurate fixation of the favorable QTL alleles.

### The population merit method

The population merit method was introduced by Lindgren and Mullin (1997) and aims to preserve the genetic variation of the breeding population by taking into account the average coancestry of the parental population. Normally, the average coancestry is calculated based on pedigree information. Unfortunately, this information is not available for both datasets. Therefore, the average genetic relationship will be used instead. The genetic relationship matrix G is calculated according to VanRaden (2008):

$$G=\frac{MM^{\prime }}{2\sum_{i=1}^{k}P_{i}\left(1-P_{i}\right)}$$ (2)

with M a matrix with *k* columns of which each column is calculated as $Z_{i}-1_{n}\left[2\left(P_{i}-0.5\right)\right]$, $Z_{i}$ the genotype of *n* individuals at the *i*-th marker, $1_{n}$ a vector of size *n* containing ones, *k* the number of markers, *n* the number of individuals in the breeding population and $P_{i}$ the frequency of the second allele at the *i*-th marker. The population merit $B_{\omega }$ is calculated as:

$$B_{\omega }={\hat{g}}_{m}-c\varphi_{\omega }$$ (3)

with ${\hat{g}}_{m}$ the mean genetic value of the parental population, *c* a penalty weight and $\varphi_{\omega }$ the average genetic relationship of the parental population. At each breeding cycle the population merit is maximized. First, 100 individuals are selected using truncation selection. Second, the mean genetic value of the parental population and the average genetic relationship are calculated. Third, the population merit is maximized iteratively by replacing each parent with another individual of the breeding population that increases the population merit. To do so, the mean genetic value of the parental population and the average genetic relationship have to be recalculated each time. The population merit is maximized when the parental population remains unchanged.

### The maximum variance total method

The maximum variance total (MVT) method aims to maximize the genetic variance of the breeding population (Cervantes *et al.* 2016). The method was developed by Bennewitz and Meuwissen (2005) and further modified by Cervantes and Meuwissen (2011). The genetic variance criterion $\text{var}\left(u_{w}\right)$ is calculated as:

$$\text{var}\left(u_{w}\right)=\frac{1}{n}\sum_{i=1}^{n}\left(\left(1+F_{i}\right)-2{\bar{G}}_{p}\right)$$ (4)

with *n* the number of selected parents, $F_{i}$ the inbreeding coefficient of parent *i* and ${\bar{G}}_{p}$ the average genetic relationship of the parents. Originally, the genetic variance criterion is calculated using the average coancestry, but due to the lack of pedigree information, the average coancestry was replaced with the average genetic relationship. Similar to the population merit method, the genetic variance is maximized iteratively. However, the MVT method does not take into account the genetic value. Therefore, it can only be used in a pre-selected population to guide the final parental selection. The MVT method will be used to select the $P_{2}$ parents from a pre-selected population similar to the scoping method. First, 300 individuals are pre-selected using truncation selection. Second, from the pre-selected individuals, 100 parents are selected using truncation selection. Finally, the $P_{2}$ parents are iteratively replaced such that the genetic variance criterion of the parental population is maximized by only using the pre-selected individuals. We expect a higher long-term gain compared with the baseline method, but a lower genetic gain compared with the scoping method.

### Training panel

The parental selection schemes are based on GEBVs that are obtained by fitting a linear mixed effects model:

$$y=1_{n}\beta +Z_{\text{TP}}u+\epsilon$$ (5)

with y a vector with phenotypic values, $1_{n}$ a vector of size *n* containing ones, *n* the number of individuals in the training panel, *β* the fixed effect (phenotypic mean), $Z_{\text{TP}}$ the incidence matrix of the training panel with marker information, u the marker effects following a normal distribution N(0,G) with $G=\sigma_{u}^{2}I_{k}$ (with $I_{k}$ the identity matrix of dimension *k*) and ϵ the residual effects following a normal distribution N(0,R) with $R=\sigma_{e}^{2}I_{n}$. Both variance components $\sigma_{u}^{2}$ and $\sigma_{e}^{2}$ are estimated by means of Restricted Maximum Likelihood (REML). The GEBVs of the individuals are calculated as:

$$\hat{g}=Z_{bc}\hat{u}$$ (6)

with $\hat{g}$ the GEBVs, $Z_{bc}$ the marker information and $\hat{u}$ the predicted marker effects. Assuming that the phenotypic data of the entire base population are available, it can be used to construct the initial TP with a total size of 764 individuals. During subsequent breeding cycles, the TP is updated with 150 new individuals selected from the breeding population, limiting the required phenotyping effort per cycle to only 150 individuals. The 150 oldest individuals of the TP are eliminated keeping the size of the TP constant. The removal of old lines in the TP does not affect the prediction accuracy significantly, but reduces the required computation time. Before training the model, markers with a minor allele frequency smaller than 0.03 are removed leading to a more accurate prediction of the GEBVs (Chang *et al.* 2018). The selection of 150 new individuals during the TP update is done using the *tails* method (Neyhart *et al.* 2017), selecting an equal number of individuals from both tails of the distribution of the GEBVs. This method delivers the highest genetic values according to Neyhart *et al.* (2017).

### Simulation of the population

The simulator was built upon the work of Neyhart *et al.* (2017), using the packages GSSimTPUpdate and hypred in R (version 3.5.2). First, the genome of barley is constructed based on marker position, allele and chromosomal information. One hundred QTL (L=100) are selected randomly from the available 1590 biallelic SNP loci. The remaining 1490 biallelic SNP loci are available as markers for prediction and selection purposes. The QTL effects are calculated according to a geometric series. At the *k*-th QTL, the favorable homozygote will have a value $a^{k}$, the heterozygote a value zero and the unfavorable homozygote a value $-a^{k}$ with a=(L−1)/(L+1). Dominance and epistasis effects were assumed to be absent. The genetic value of an individual is calculated as the sum of all present QTL alleles. Different variables are calculated to track the fixation of QTL alleles. The maximum genetic value is the sum of the favorable QTL effects. The fixed genetic value is the sum of the QTL effects that are fixed. The maximum reachable genetic value is the sum of the QTL effects that are fixed (both favorable and unfavorable) and the sum of the favorable QTL effects that are not yet fixed. It represents the maximum genetic value that could still be reached, taking into account the fixation of unfavorable QTL alleles. All these variables are converted into a percentage, where the maximum genetic value of 1 can only be achieved if all favorable QTL alleles are present. The phenotypic values are calculated as follows:

$$y_{ij}=g_{i}+e_{j}+\epsilon_{ij}$$ (7)

with $y_{ij}$ the phenotypic value of the *i*-th individual in the *j*-th environment, $g_{i}$ the genetic value of the *i*-th individual, $e_{j}$ the *j*-th environmental effect and $\epsilon_{ij}$ the residual effect of the *i*-th individual and the *j*-th environment. Three different environmental effects are drawn from a normal distribution with mean 0 and a variance component $\sigma_{E}^{2}$ which is defined as eight times the genetic variance (Bernardo 2014). The residual effect is drawn from a normal distribution with mean 0 and a variance component $\sigma_{R}^{2}$, with $\sigma_{R}^{2}$ scaled to simulate a population with a heritability $\left(h^{2}\right)$ of 0.5. The phenotypic value of each individual is the average value over the different environments.

### Data availability

The scripts, figures, datasets of the base population and supplementary data are available from the github repository https://github.com/biointec/scoping. The dataset and the simulation of the recurrent breeding cycle have been reported and published by Neyhart *et al.* (2017) (https://doi:10.1534/g3.117.040550).

## Results

### The baseline method

**Figure 2:**
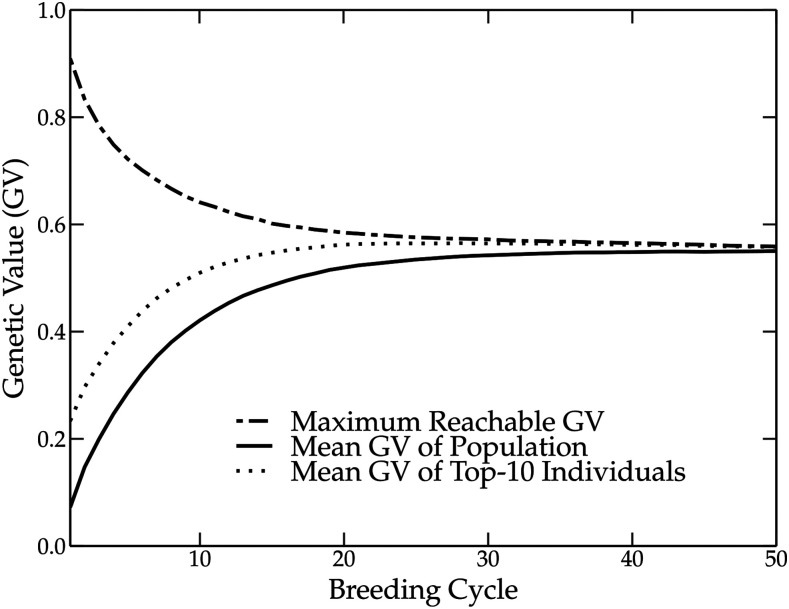
Simulation results using the baseline method over 50 breeding cycles. The mean genetic value of the breeding population increases rapidly over the first breeding cycles. The truncation selection, however, causes the loss of several favorable QTL alleles, reducing the maximum reachable genetic value and causing a premature convergence of the genetic value to a local optimum. The top-10 individuals of the population have a higher mean genetic value than the breeding population, but after several breeding cycles, the genetic variation is reduced, closing the gap between the top-10 individuals and the rest of the breeding population.

The baseline method combines truncation selection with random mating. Our results are similar to those reported by Neyhart *et al.* (2017). During the first 10 to 20 breeding cycles, we observe a steep increase in genetic value and rapid fixation of QTL alleles (see Figure 2). The maximum reachable genetic value is reduced by more than 40%, due to the loss of favorable QTL alleles in the breeding population. It is interesting to also consider the mean genetic value of the 10 individuals with the highest genetic values. Those individuals are of particular interest to breeders for commercialization purposes. Therefore, their genetic value is more important than the mean genetic value of the breeding population. In the baseline method, the top-10 individuals have a higher genetic value over the first breeding cycles, but due to strong fixation, the genetic variation is reduced and the difference between the top individuals and the breeding population average becomes smaller.

### The scoping method

**Figure 3:**
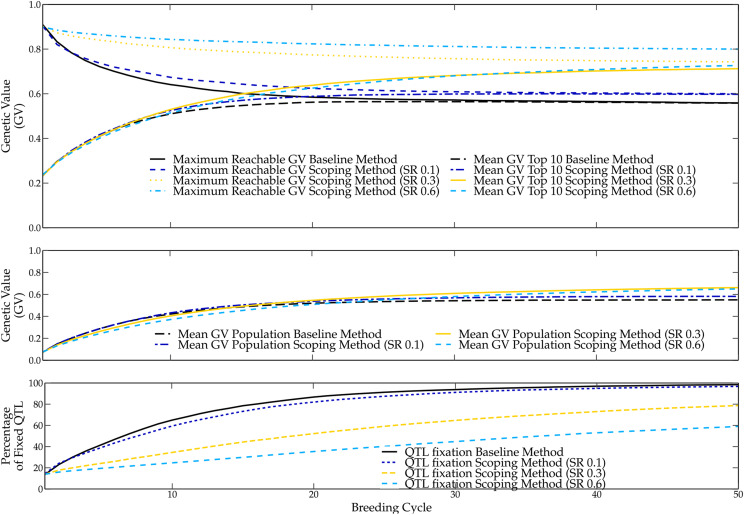
Simulation results using the scoping method for an SR of 0.1, 0.3 and 0.6, simulated over 50 breeding cycles. Additionally, the results of the baseline method are shown for the sake of comparison. In the top figure, the mean genetic value of the top-10 individuals and the maximum reachable genetic value are shown for different SR values and the baseline method. In the middle figure, the mean genetic value of the breeding population is shown for different SR values and the baseline method. In the bottom figure, the rate of QTL fixation is shown for different SR values and the baseline method.

The scoping method introduces the scoping rate (SR) as a new parameter. With the SR, the breeder can control what fraction of the upper tail of the GEBV distribution will be considered for parental selection. Using a small SR, only individuals with high GEBVs will be considered, leading to truncation selection. When a higher SR is used instead, individuals with lower GEBVs will also be considered as candidates, making it possible to preserve the genetic variation of the breeding population. The SR provides the breeder with the option to choose between the maximization of the rate of genetic progression in the short term on the one hand or the maximization of the genetic variation in the long term on the other hand. As expected, the scoping method yields somewhat lower mean genetic values over the first ten breeding cycles (see Figure 3). However, the mean genetic value of the top-10 individuals is only slightly lower compared with the baseline method. Certainly, for small SR values (0.1 to 0.3) the difference in genetic value is negligible.

After the tenth breeding cycle, the loss of several favorable QTL alleles causes the baseline method to reach a local optimum, rendering it less efficient than the scoping method. In contrast, by preserving the genetic variation within the breeding population, the scoping method strongly reduces the loss of favorable QTL alleles, thus preserving the potential to reach high genetic values. A higher SR will better prevent the loss of favorable QTL alleles, however, due to a slower increase in genetic value, a high SR will require a longer time before outperforming the baseline method. Therefore, the use of a smaller SR is preferred. It delivers high genetic values in both the short and the long term.

The SR of 0.1 is a special case as it results in the same parental selection as the baseline method, but it uses an alternative mating design to maximize the genetic variation of the offspring. After 50 breeding cycles, this leads to a 4 percentage points higher mean genetic value of the top-10 individuals in favor of the scoping method. This demonstrates that maximizing the genetic variation increases the genetic value in the long term. The SR of 0.3 yields high genetic values in both the short and the long term. Only eight breeding cycles are needed before the top-10 individuals outperform the baseline method. Over those eight breeding cycles, the difference in genetic value between the baseline method and the scoping method are negligible. After 12 breeding cycles, the mean genetic value of the population surpasses that of the baseline method. Ultimately, after 50 breeding cycles, the scoping method with an SR of 0.3 yields a mean genetic value of 0.71 over the top-10 individuals, a 15 percentage points increase compared with the baseline method.

### The population merit method

**Figure 4:**
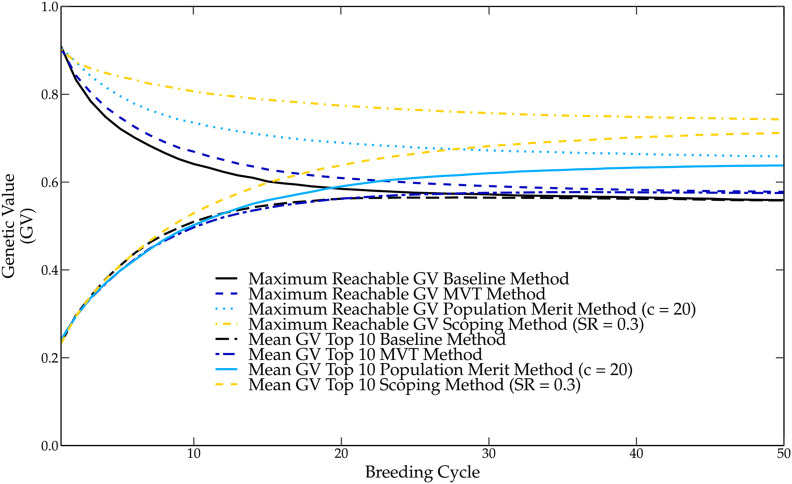
The genetic value of the different parental selection methods over 50 breeding cycles. The genetic value in the long term is the lowest when using the baseline method, followed by the maximum value total (MVT) method, population merit method and the scoping method, which delivers the highest genetic values in the long term.

The population merit method preserves the genetic variance by reducing the average genetic relationship of the parental population, leading to a higher genetic gain in the long term compared with the baseline method (see Figure 4). Despite the fact that a higher long-term gain is observed, the population merit method only retains a fraction of the genetic variation, still causing the fixation of several unfavorable QTL alleles and a premature convergence of the genetic value to a local optimum. Compared to the scoping method, the same genetic value is observed over the first eight breeding cycles. However, the population merit method causes a strong reduction in the maximum reachable genetic value rendering this method less efficient in the long term than the scoping method. Several values for the penalty weight *c* were tested and the best results for c=20 are reported. At breeding cycle 50, only an 8 percentage points increase in the genetic value was observed for the population merit method compared with the baseline method, while a 15 percentage points increase in genetic value was observed for the scoping method.

### The maximum variance total method

The MVT method combines the average genetic relationship and the average inbreeding coefficient to maximize the genetic variation of the breeding population. This method was used to compare the mating design of the scoping method with the MVT method by using the same pre-selected population to select the $P_{2}$ parents. Only a small increase of the genetic gain was observed for the MVT method compared with the baseline method (see Figure 4). Using a pre-selected population combined with a truncation selection of the $P_{1}$ parents, the MVT method only preserved a small part of the genetic variation compared with the scoping method, causing the loss of several favorable QTL alleles and thus reducing the maximum reachable genetic value. At breeding cycle 50, only a 2 percentage points higher genetic value was observed compared with the baseline method, rendering this method less efficient than the scoping method.

The mean genetic value of the breeding population, mean genetic value of the top-10 individuals and the maximum reachable genetic value of all the proposed methods are reported in Table S1, Table S2 and Table S3, respectively.

## Discussion

### Risks of truncation selection

**Figure 5:**
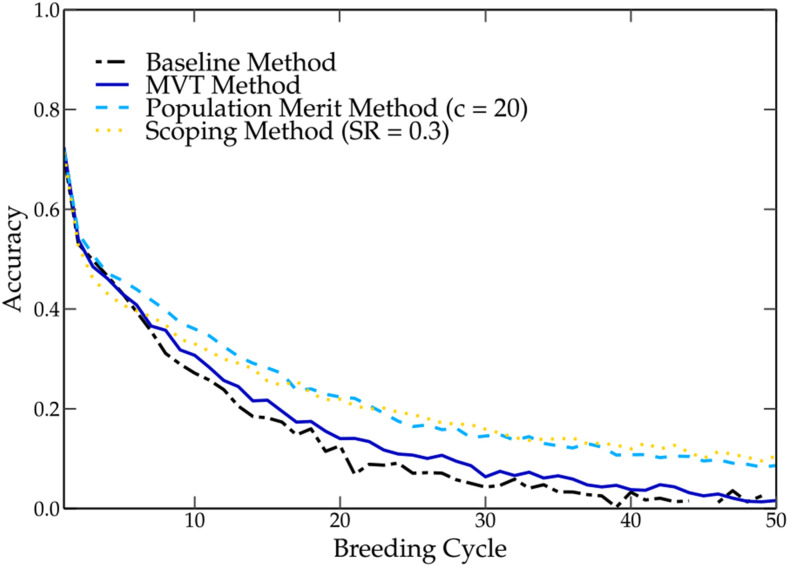
The genetic accuracy of the different parental selection methods over 50 breeding cycles. The genetic accuracy drops the fastest when using the baseline method, followed by the maximum variance total (MVT) method, population merit method and the scoping method with an SR of 0.3.

**Figure 6:**
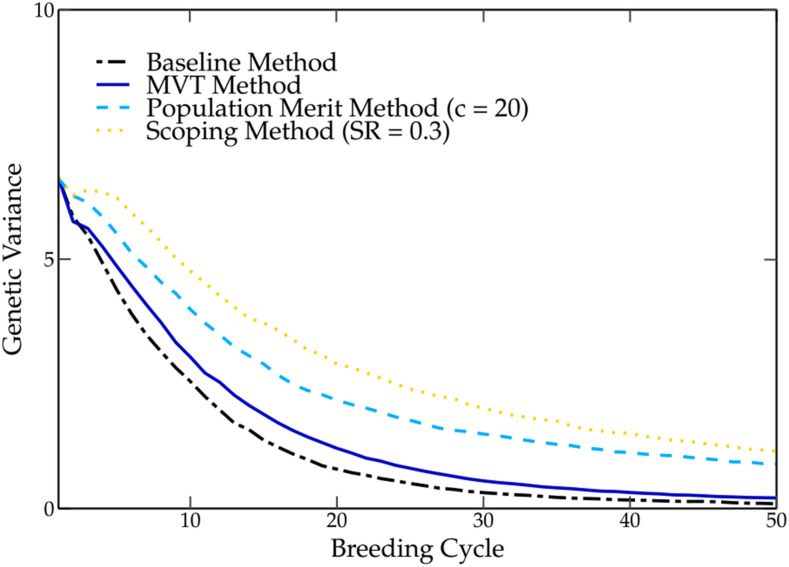
The genetic variance of the different parental selection methods over 50 breeding cycles. The genetic variance drops the fastest when using the baseline method, followed by the maximum variance total (MVT) method, population merit method and the scoping method with an SR of 0.3.

Nowadays, the use of truncation selection is still popular among breeders, despite the fact that fixation of unfavorable QTL alleles associated with this selection method has been reported (Jannink 2010). By selecting parents based on their GEBVs using truncation selection, breeders hope to maximally pass favorable QTL alleles on to the next generation. However, the GEBV represents only a single value per individual that integrates the genetic information of more than 1000 molecular markers (see Equation (6)). In contrast to MAS, in genomic selection, only a fraction of those molecular markers are in strong linkage disequilibrium with QTL (Meuwissen *et al.* 2001). By summarizing the information of all those marker effects into a single number, important genetic information is lost, rendering it difficult to detect the presence or absence of favorable QTL alleles. This is especially the case when rare marker effects are masked by the presence of many other marker effects. This was demonstrated in the baseline method, where several negative QTL alleles were fixed in the breeding population. Eynard *et al.* (2017) simplified the selection of favorable QTL alleles by assigning weights to rare marker alleles. Nevertheless, it is clear that truncation selection does not guarantee the presence of all favorable QTL alleles in the parental population and could hence result in their loss. However, the baseline method has a positive genetic gain over each breeding cycle, indicating that the fixation of the favorable QTL alleles has a higher impact on the genetic value than the fixation of unfavorable QTL alleles. The reduction of the genetic variation of the breeding population, which is often associated with truncation selection, causes a reduction in prediction accuracy (Heffner *et al.* 2009), which implies poorly estimated marker effects and substandard parental selections (see Figure 5). In turn, a poor parental selection in combination with a low genetic variation will further contribute to the loss of favorable QTL alleles as observed in the baseline method. Jannink (2010) tackled this problem by limiting the rate of inbreeding in the TP and thereby reducing the loss of genetic variation. However, methods based on truncation selection still cause the loss of several favorable QTL alleles. The scoping method also tackles this problem by preserving the genetic variation throughout the breeding cycles and increases long-term gain (see Figure 6).

### Preserving genetic variation for long-term benefits

Truncation selection causes the loss of several favorable and unfavorable QTL alleles, reducing the genetic variation of the breeding population and causing a premature convergence of the genetic value to a local optimum. Reintroducing new semi-wild species can temporally increase the genetic variation. However, those semi-wild species need several cycles of pre-breeding making this approach less cost and time efficient. The scoping method *consistently* preserves the genetic variation in the breeding population for as long as possible and thus avoids a premature convergence of the genetic value.

The scoping method does not only preserve the genetic variation in the breeding population but also in the TP, leading to an improved prediction accuracy (see Figures 6 and 5) (Voss-Fels *et al.* 2018). In the case of the scoping method, by preserving both marker alleles at each marker, both alleles at each QTL were also preserved in the breeding population. If certain marker effects were masked or poorly predicted, the alternate allele could still be built into the next offspring.

The scoping method delivers an important message. Fixation of favorable QTL alleles is not a prerequisite to obtain high genetic values. The scoping method was able to outperform the baseline method with only 40% of the QTL alleles fixed in the breeding population. Preserving both alleles at each QTL prevents the elimination of poorly predicted QTL alleles.

### Comparison of the scoping method With existing methods

In this paper two existing methods (the population merit method (Lindgren and Mullin 1997) and the MVT method (Cervantes *et al.* 2016)) were compared with the scoping method. The population merit method calculates a score per parental population which is maximized using an iterative algorithm. By penalizing a high genetic relationship between parents, the loss in genetic variation is minimized. The population merit method delivered a significant improvement compared with the baseline method, but the scoping method was able to outperform the population merit method within the first 10 breeding cycles. The genetic relationship matrix alone was not enough to preserve the genetic variation in the breeding population. Over the first breeding cycles, a strong decrease in the maximum genetic value was observed, indicating the fixation of several unfavorable QTL alleles. This was probably caused by the loss in genetic variation, leading to a lower prediction accuracy and thus a poor estimation of the additive marker effects (see Figures 6 and 5).

The population merit method reduces the information of the genetic relationship matrix into a single averaged value. This certainly helps to preserve the genetic variation but it does not guarantee that all the marker alleles will be preserved in the breeding population. A decrease of the maximum reachable genetic value is a good indicator to monitor the loss of favorable QTL alleles. A good parental selection method should be able to keep the maximum reachable genetic value fixed. It is clear that the population merit method fails in preventing the loss of those favorable QTL alleles. The scoping method includes a Boolean vector that ensures the inclusion of all available marker alleles, reducing the loss of favorable QTL alleles and thus maximizing the genetic variation over each breeding cycle.

The MVT method does not take into account the genetic value of a current parental population. The method should maximize the genetic variance, but because the genetic value is not included in the selection criterion, no high genetic gain can be obtained in the short or long term. Therefore, the MVT method was combined with a pre-selection and truncation selection of the $P_{1}$ parents. Both the scoping and MVT methods used the same pool of pre-selected individuals to select the parental population. The scoping method delivered a higher genetic gain compared with the MVT method. Again, reducing the information of the genetic relationship matrix into a single number reduces the available information, causing a lower genetic variation compared with the scoping method.

It is clear that the scoping method can better preserve the genetic variation of the breeding population. The use of the inbreeding coefficient and relationship matrix only prevents the selection of closely related individuals, but it does not directly prevent the loss of certain QTL alleles. The scoping method will always try to reduce the loss of favorable QTL alleles by preserving both marker alleles in the breeding population. This has proven to be the most successful method, delivering the highest genetic values in the long term.

### Robustness of the scoping method

**Figure 7:**
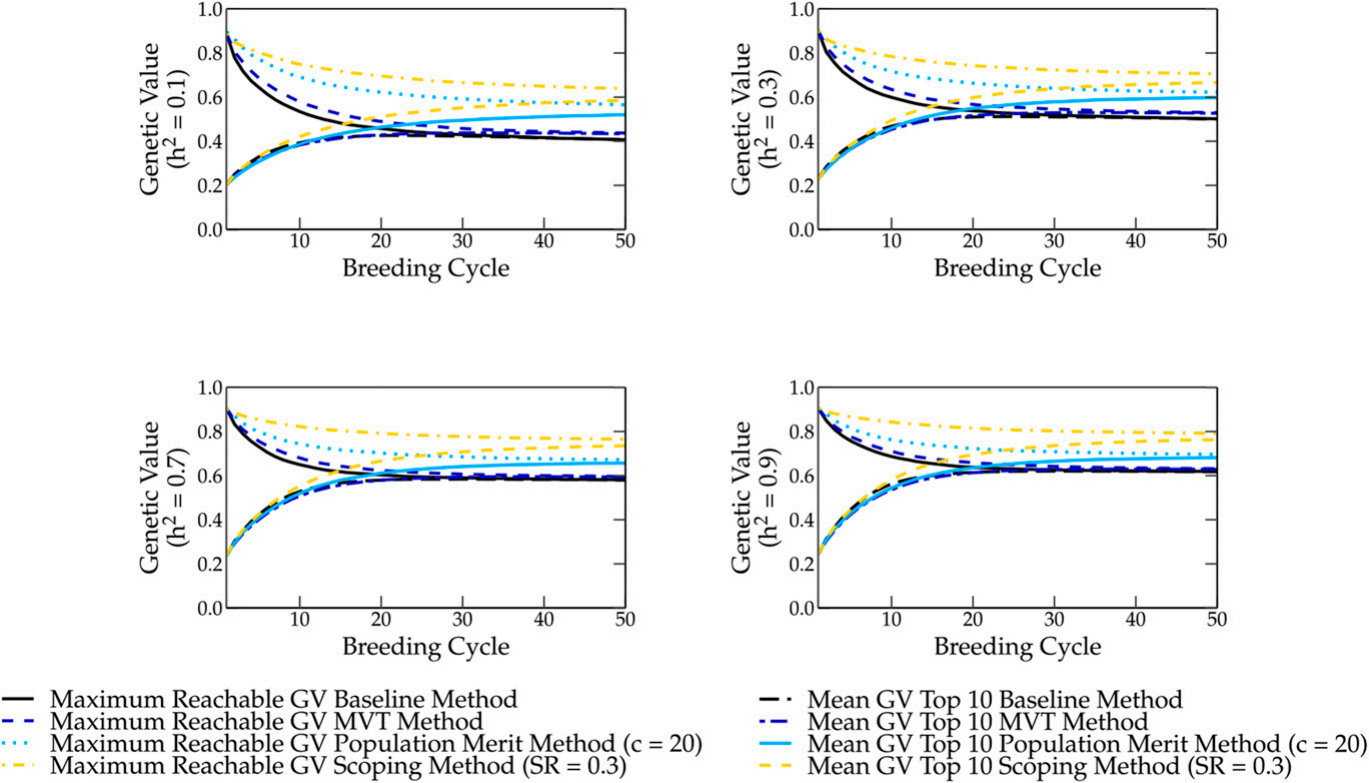
The genetic value of the different parental selection methods for different heritabilities over 50 breeding cycles. The genetic value in the long term is the lowest when using the baseline method, followed by the maximum value total (MVT) method, population merit method and the scoping method, which delivers the highest genetic values in the long term.

The robustness of the scoping method has been tested and compared with the baseline, population merit and MVT methods using different genome constructions. We have compared the different methods for a heritability of 0.1, 0.3, 0.7 and 0.9 (see Figure 7). The genetic value was also studied for 50 (see Figure S1) and 200 QTL (see Figure S2).

## Conclusion

In our simulation study, we demonstrated the need for an alternative parental selection method to prevent the convergence of the genetic value of the breeding population to a local optimum caused by the loss of favorable QTL alleles. Truncation parental selection leads to a rapid fixation, but also to the loss of several favorable QTL alleles, causing the convergence of the genetic values to a suboptimal value and reducing the possibility to reach the global optimum in the long term. Consistently preserving the genetic variation (scoping method) leads to higher genetic values in the long term and only a slightly lower genetic value in the short term.

## References

[bib1] Akdemir D., and Sánchez J. I., 2016 Efficient breeding by genomic mating. Front. Genet. 7: 210 10.3389/fgene.2016.00210 27965707 PMC5126051

[bib2] Akdemir D., Sanchez J. I., and Jannink J. L., 2015 Optimization of genomic selection training populations with a genetic algorithm. Genet. Sel. Evol. 47: 38 10.1186/s12711-015-0116-6 25943105 PMC4422310

[bib3] Bennewitz J., and Meuwissen T. H., 2005 A novel method for the estimation of the relative importance of breeds in order to conserve the total genetic variance. Genet. Sel. Evol. 37: 315–337. 10.1186/1297-9686-37-4-315 15823238 PMC2697237

[bib4] Bernardo R., 2014 Genomewide selection of parental inbreds: Classes of loci and virtual biparental populations. Crop Sci. 54: 2586–2595. 10.2135/cropsci2014.01.0088

[bib5] Bernardo R., and Yu J., 2007 Prospects for genomewide selection for quantitative traits in maize. Crop Sci. 47: 1082–1090. 10.2135/cropsci2006.11.0690

[bib6] Beyene Y., Semagn K., Mugo S., Tarekegne A., Babu R., 2015 Genetic gains in grain yield through genomic selection in eight bi-parental maize populations under drought stress. Crop Sci. 55: 154–163. 10.2135/cropsci2014.07.0460

[bib7] Brisbane J. R., and Gibson J. P., 1995 Balancing selection response and inbreeding by including predicted stabilised genetic contributions in selection decisions. Genet. Sel. Evol. 27: 541–549. 10.1186/1297-9686-27-6-541

[bib8] Cervantes I., Gutiérrez J. P., and Meuwissen T. H., 2016 Response to selection while maximizing genetic variance in small populations. Genet. Sel. Evol. 48: 69 10.1186/s12711-016-0248-3 27649906 PMC5030739

[bib9] Cervantes I., and Meuwissen T. H., 2011 Maximization of total genetic variance in breed conservation programmes. J. Anim. Breed. Genet. 128: 465–472. 10.1111/j.1439-0388.2011.00923.x 22059580

[bib10] Chang L. Y., Toghiani S., Ling A., Aggrey S. E., and Rekaya R., 2018 High density marker panels, SNPs prioritizing and accuracy of genomic selection. BMC Genet. 19: 1–10. 29304753 10.1186/s12863-017-0595-2 PMC5756446

[bib11] Cros D., Tchounke B., and Nkague-Nkamba L., 2018 Training genomic selection models across several breeding cycles increases genetic gain in oil palm in silico study. Mol. Breed. 38: 89 10.1007/s11032-018-0850-x

[bib12] Crossa J., De Los Campos G., Pérez P., Gianola D., Burgueño J., 2010 Prediction of genetic values of quantitative traits in plant breeding using pedigree and molecular markers. Genetics 186: 713–724. 10.1534/genetics.110.118521 20813882 PMC2954475

[bib13] Daetwyler H. D., Hayden M. J., Spangenberg G. C., and Hayes B. J., 2015 Selection on optimal haploid value increases genetic gain and preserves more genetic diversity relative to genomic selection. Genetics 200: 1341–1348. 10.1534/genetics.115.178038 26092719 PMC4574260

[bib14] de Roos A. P. W., Hayes B. J., Spelman R. J., and Goddard M. E., 2008 Linkage disequilibrium and persistence of phase in Holstein-Friesian, Jersey and Angus cattle. Genetics 179: 1503–1512. 10.1534/genetics.107.084301 18622038 PMC2475750

[bib15] Dekkers J. C., and Hospital F., 2002 The use of molecular genetics in the improvement of agricultural populations. Nat. Rev. Genet. 3: 22–32. 10.1038/nrg701 11823788

[bib16] Eynard S. E., Croiseau P., Laloë D., Fritz S., Calus M. P. L., 2017 Which Individuals To Choose To Update the Reference Population? Minimizing the Loss of Genetic Diversity in Animal Genomic Selection Programs. G3 (Bethesda) 8: 113–121. 10.1534/g3.117.1117 PMC5765340 29133511

[bib17] Goddard M. E. and Hayes B. J., 2002 Optimisation of response using molecular data. Proc. 7th world Congr. Appl. Livest. Prod. Montpellier, France, August 19–23, 2002

[bib18] Gorjanc G., Gaynor R. C., and Hickey J. M., 2018 Optimal cross selection for long-term genetic gain in two-part programs with rapid recurrent genomic selection. Theor. Appl. Genet. 131: 1953–1966. 10.1007/s00122-018-3125-3 29876589 PMC6096640

[bib19] Hayes B. J., Bowman P. J., Chamberlain A. J., and Goddard M. E., 2009 Invited review: Genomic selection in dairy cattle: progress and challenges. J. Dairy Sci. 92: 433–443. 10.3168/jds.2008-1646 19164653

[bib20] Heffner E. L., Lorenz A. J., Jannink J. L., and Sorrells M. E., 2010 Plant breeding with Genomic selection: Gain per unit time and cost. Crop Sci. 50: 1681–1690. 10.2135/cropsci2009.11.0662

[bib21] Heffner E. L., Sorrells M. E., and Jannink J. L., 2009 Genomic selection for crop improvement. Crop Sci. 49: 1–12. 10.2135/cropsci2008.08.0512

[bib22] Jannink J. L., 2010 Dynamics of long-term genomic selection. Genet. Sel. Evol. 42: 35 10.1186/1297-9686-42-35 20712894 PMC2936280

[bib23] Juliana P., Singh R. P., Poland J., Mondal S., Crossa J., 2018 Prospects and challenges of applied genomic selection––a new paradigm in breeding for grain yield in bread wheat. Plant Genome 11: 1–17. 10.3835/plantgenome2018.03.0017 PMC7822054 30512048

[bib24] Lehermeier C., Teyssèdre S., and Schön C. C., 2017 Genetic gain increases by applying the usefulness criterion with improved variance prediction in selection of crosses. Genetics 207: 1651–1661. 29038144 10.1534/genetics.117.300403 PMC5714471

[bib25] Lindgren D., and Mullin T. J., 1997 Balancing gain and relatedness in selection. Silvae Genet. 46: 124–129.

[bib26] Liu H., Meuwissen T. H., Sørensen A. C., and Berg P., 2015 Upweighting rare favourable alleles increases long-term genetic gain in genomic selection programs. Genet. Sel. Evol. 47: 19 10.1186/s12711-015-0101-0 25886296 PMC4367977

[bib27] Meuwissen T., Hayes B., and Goddard M., 2001 Prediction of total genetic value using genome-wide dense marker maps. Genetics 157: 1819–1829. 11290733 10.1093/genetics/157.4.1819 PMC1461589

[bib28] Müller D., Schopp P., and Melchinger A. E., 2018 Selection on expected maximum haploid breeding values can increase genetic gain in recurrent genomic selection. G3 (Bethesda) 8: 1173–1181. 29434032 10.1534/g3.118.200091 PMC5873908

[bib29] Neyhart J. L., Tiede T., Lorenz A. J., and Smith K. P., 2017 Evaluating Methods of Updating Training Data in Long-Term Genomewide Selection. G3 (Bethesda) 7: 1499–1510. 28315831 10.1534/g3.117.040550 PMC5427505

[bib30] Rincent R., Laloë D., Nicolas S., Altmann T., Brunel D., 2012 Maximizing the reliability of genomic selection by optimizing the calibration set of reference individuals: Comparison of methods in two diverse groups of maize inbreds (Zea mays L.). Genetics 192: 715–728. 10.1534/genetics.112.141473 22865733 PMC3454892

[bib31] Sun J., Poland J. A., Mondal S., Crossa J., Juliana P., 2019 High-throughput phenotyping platforms enhance genomic selection for wheat grain yield across populations and cycles in early stage. Theor. Appl. Genet. 132: 1705–1720. 10.1007/s00122-019-03309-0 30778634

[bib32] Suontama M., Klápště J., Telfer E., Graham N., Stovold T., 2019 Efficiency of genomic prediction across two Eucalyptus nitens seed orchards with different selection histories. Heredity 122: 370–379. 10.1038/s41437-018-0119-5 29980794 PMC6460750

[bib33] Tester M., and Landridge P., 2010 Breeding Technologies to Increase Crop Production in a Changing World. Science 327: 818–822. 10.1126/science.1183700 20150489

[bib34] VanRaden P. M., 2008 Efficient Methods to Compute Genomic Predictions. J. Dairy Sci. 91: 4414–4423. 10.3168/jds.2007-0980 18946147

[bib35] VanRaden P. M., Van Tassell C. P., Wiggans G. R., Sonstegard T. S., Schnabel R. D., 2009 Invited review: Reliability of genomic predictions for North American Holstein bulls. J. Dairy Sci. 92: 16–24. 10.3168/jds.2008-1514 19109259

[bib36] Voss-Fels K. P., Cooper M., and Hayes B. J., 2018 Accelerating crop genetic gains with genomic selection. Theor. Appl. Genet. 132: 669–686. 10.1007/s00122-018-3270-8 30569365

[bib37] Wang Q., Yu Y., Zhang Q., Zhang X., Huang H., 2019 Evaluation on the genomic selection in Litopenaeus vannamei for the resistance against Vibrio parahaemolyticus. Aquaculture 505: 212–216. 10.1016/j.aquaculture.2019.02.055

